# Production of β-Cyclocitral and Its Precursor β-Carotene in *Microcystis aeruginosa*: Variation at Population and Single-Cell Levels

**DOI:** 10.3390/toxins14030201

**Published:** 2022-03-09

**Authors:** Xuejian Wang, Yinjie Zhu, Delin Hou, Fei Teng, Zhonghua Cai, Yi Tao

**Affiliations:** 1 Groundwater Provincial Engineering Research Center for Urban Water Recycling and Environmental Safety, Shenzhen International Graduate School, Tsinghua University, Shenzhen 518055, China; wxj17@tsinghua.org.cn (X.W.); zhu-yj21@mails.tsinghua.edu.cn (Y.Z.); delinhou1990@163.com (D.H.); 2 Key Laboratory of Microorganism Application and Risk Control (MARC) of Shenzhen, Shenzhen International Graduate School, Tsinghua University, Shenzhen 518055, China; teng.fei@sz.tsinghua.edu.cn; 3 The Division of Ocean Science and Technology, Shenzhen International Graduate School, Tsinghua University, Shenzhen 518055, China; caizh@sz.tsinghua.edu.cn

**Keywords:** cyanobacteria, *Microcystis aeruginosa*, β-cyclocitral, growth phase, cellular quota, β-carotene

## Abstract

Bloom-forming cyanobacteria produce and release odorous compounds and pose threats to the biodiversity of aquatic ecosystem and to the drinking water supply. In this study, the concentrations of β-cyclocitral in different bacterial growth phases were investigated using GC–MS to determine the growth stage of *Microcystis aeruginosa* at high risk for β-cyclocitral production. Moreover, the synchronicity of the production of β-cyclocitral and its precursor β-carotene at both population and single-cell levels was assessed. The results indicated that β-cyclocitral was the main odorous compound produced by *M. aeruginosa* cells. The intracellular concentration of β-cyclocitral (*C*_β-cc_) as well as its cellular quota (*Q*_β-cc_) increased synchronously in the log phase, along with the increase of cell density. However, they reached the maximum values of 415 μg/L and 10.7 fg/cell in the late stationary phase and early stationary phase, respectively. The early stage of the stationary phase is more important for β-cyclocitral monitoring, and the sharp increase in *Q*_β-cc_ is valuable for anticipating the subsequent increase in *C*_β-cc_. The molar concentrations of β-cyclocitral and β-carotene showed a linear relationship, with an R^2^ value of 0.92, suggesting that the production of β-cyclocitral was linearly dependent on that of β-carotene, especially during the log phase. However, the increase in *Q*_β-cc_ was slower than that in β-carotene during the stationary phase, suggesting that β-cyclocitral production turned to be carotene oxygenase-limited when the growth rate decreased. These results demonstrate that variations of β-cyclocitral production on a single-cell level during different bacterial growth phases should be given serious consideration when monitoring and controlling the production of odorous compounds by *M. aeruginosa* blooms.

## 1. Introduction

Cyanobacteria blooms are notorious worldwide for interrupting the supply of water for drinking, irrigation, and sanitation [1,2,3]. The production of secondary metabolites of cyanobacteria, including toxic and odorous compounds, poses a great risk to human daily life and even causes health problems [4]. Toxins produced by several cyanobacterial genera, including *Anabaena*, *Aphanizomenon*, *Microcystis*, and *Planktothrix*, cause skin illness, liver cancer, and even death of human beings [5]. In 2011, a record-setting cyanobacteria bloom was experienced in Lake Erie [6], with consequent concentrations of microcystin as high as 4500 μg/L, the exposure to which has subsequently been of growing concern [7]. The occurrence of odorous compounds in drinking water is always unpleasant for consumers [8,9,10], though not necessarily posing risks to human health. In 2007, two million residents in Wuxi City, China, suffered a 14 days’ water cut-off, owing to the highly unpleasant odor of drinking water caused by severe cyanobacteria blooms in Lake Taihu [7,11].

As the most widespread and health-threatening bloom-forming cyanobacteria species [12], *M. aeruginosa* produces β-cyclocitral, a main odorous compound fouling algae-laden water with a sweet-tobacco and grape odor [13], which can be perceived at β-cyclocitral concentrations as low as 0.5 μg/L [14]. β-cyclocitral from *M. aeruginosa* is the oxidation product of β-carotene by either carotene oxygenase [15] or reactive oxygen species (ROS) via a non-enzymatic pathway [16]. For the enzymatic pathway, carotene oxygenase cleaves β-carotene specially at positions 7, 8, and 7′, 8′ without affecting echinenone and myxoxanthophyll, forming β-cyclocitral and crocetindial [15]. For the non-enzymatic pathway, for example, under high light stress and temperature, elevated ROS levels including ^1^O_2_ induce a decrease in intracellular β-carotene and the release of β-cyclocitral [16,17]. It has been proposed that β-cyclocitral plays a role as a defense signal that indicates poor-quality food to grazers such as *Daphnia* [14]. Moreover, it has been reported that β-cyclocitral has a lytic effect on cyanobacteria cells [18], which leads to a characteristic blue color of water [19].

There have been previous studies that revealed the existence of β-cyclocitral in high concentrations in natural water bodies [20,21,22,23,24]. It is noteworthy that β-cyclocitral concentrations in Lake Taihu varied significantly in different studies [22,23,24]. This was probably due to spatial and temporal variations of this compound’s levels across this lake, resulting from the effect of different environmental factors, including water temperature and nutrient concentrations [25].

The concentrations of β-cyclocitral and β-carotene were found to be correlated to the growth phase of *M. aeruginosa* in the lab [26]. In a study, during a 50-day incubation, the highest concentration of β-cyclocitral was 2000 μg/L, with intracellular β-cyclocitral consisting of more than 99% of total β-cyclocitral; the concentration of β-cyclocitral per cell (i.e., the cellular quota, *Q*_β-cc_) ranged from 41 to 865 fg [26]. Additionally, a strong correlation between β-carotene and β-cyclocitral concentrations (R^2^ = 0.96) was observed during all growth stages [26]. However, another study indicated that β-cyclocitral production capacity (i.e., *Q*_β-cc_) was independent of algal cell age during the stationary phase [14]. It was also claimed in previous studies that N or P limitation could boost the emission of β-cyclocitral [27,28]. However, the production kinetics of β-cyclocitral at both population and single-cell levels remains unclear. Moreover, the production synchronization of β-cyclocitral and its precursor, β-carotene, requires further investigation.

In this study, the production of β-cyclocitral was detected during a complete cycle of growth and replication, and β-cyclocitral quota was calculated to characterize the production in different growth phases. Photosynthetic activity and pigments were also examined to understand the effects of energy supply and metabolic activity on the odor-producing process. In addition, the relationship between β-cyclocitral and its precursor β-carotene and the changing trend in the cellular quotas of β-cyclocitral and β-carotene were explored and discussed.

## 2. Results

### 2.1. Growth Characteristics of M. aeruginosa

**Figure 1:**
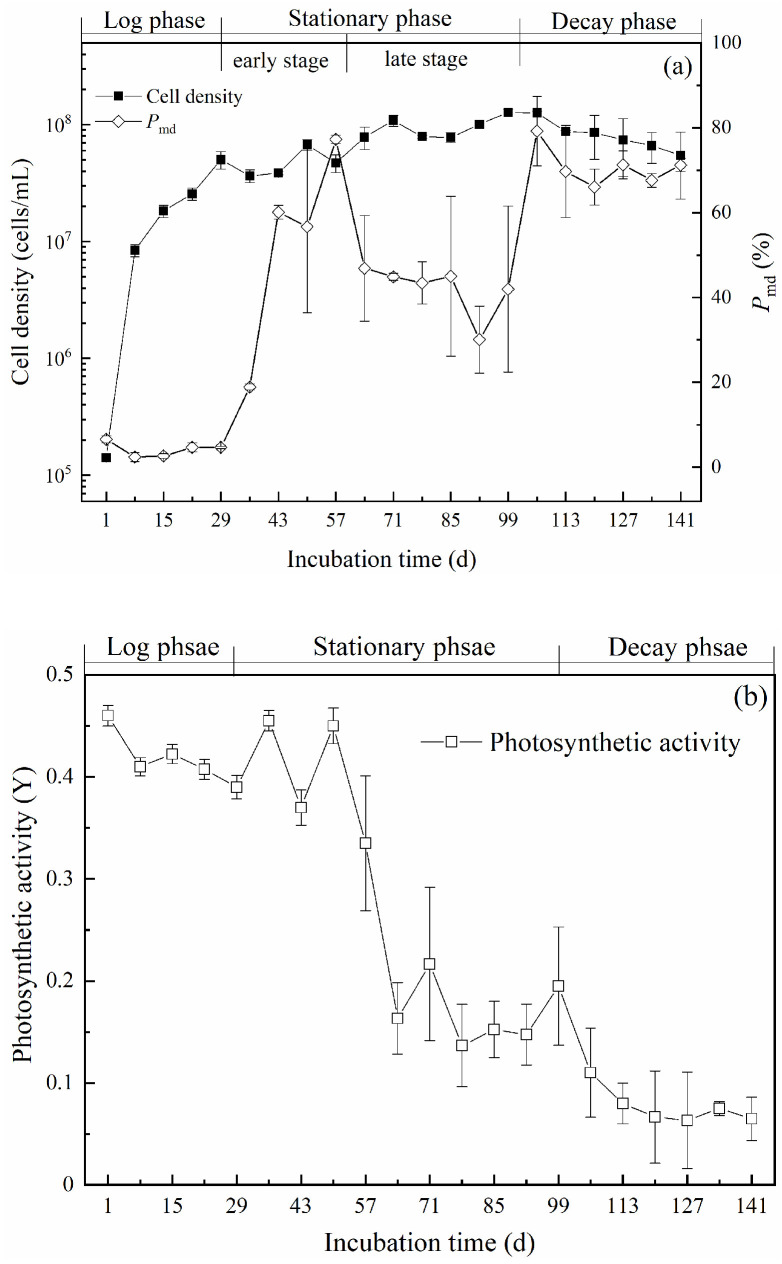
Cell density, percentage of membrane-damaged cells (*P*_md_) (**a**), and photosynthetic activity (**b**) of *Microcystis aeruginosa* cells during incubation.

Variations of cell density, membrane integrity, and photosynthetic activity during culturing are shown in Figure 1. As shown in Figure 1a, cell density rapidly increased from the initial value of 1.4 × 10^5^ cells/mL to 5.0 × 10^7^ cells/mL on the 29th day of incubation, indicating a typical logarithmic growth period with a 356-time increase. During the log phase, the percentage of dead cells, represented by the *P*_md_ value, remained below 6.6%, which confirmed healthy growth conditions for the cells. During the period from the 29th to the 99th day, cell density continuously increased, with a significant lower growth rate (Figure S1 in the Supplementary Materials), and reached the maximum value of 1.3 × 10^8^ cells/mL on the 99th day. The *P*_md_ value rose significantly and varied within 30% and 80%. The phenomenon of a constant cell density with continuous division of some cells and gradually emerging cell death suggested a stationary growth phase within the 29th and the 99th day. Moreover, the *P*_md_ value was higher between the 29th and the 57th day than in the subsequent period between the 57th and the 99th day. Thus, the stationary phase could be divided into an early stage (29th–57th day) and a late stage (57th–99th day). Cell density began to decrease after the 99th day and declined to 5.5 × 10^7^ cells/mL on the 141st day. More importantly, most algal cells were broken with a sharp increase in the *P*_md_ value, which increased to 79% on the 106th day and remained constant at approximately 70%. The death of algal cells markedly exceeded the proliferation of new ones, indicating the achievement of the decay phase from the 99th to 141st day. The lag phase was not obvious in this study, as the initially inoculated cells were in the logarithmic period.

Variation of the photosynthetic activity of *M. aeruginosa* cells is shown in Figure 1b. In the logarithmic period, Y was stable, at approximately 0.4, which indicated the cells had a strong photosynthetic activity. Between the 29th and the 57th day, the Y value fluctuated greatly above 0.34. However, during the period between the 57th and the 99th day, the Y value plunged to 0.16 on the 64th day and remained constant at about 0.15 afterwards. The results showed a significant reduction in photosynthetic activity and therefore verified the division of the stationary phase. The photosynthetic pigments in *M. aeruginosa* cells decreased significantly in the stationary phase (Supplementary Figure S2). The decline in photons reception might be the main reason for the rapid decrease in photosynthetic activity. In addition, the subsequent stabilization of photosynthetic activity might be the key point for maintaining the stability of algal cell density. The lower photosynthetic activity could not provide sufficient energy for growth and thus induced a marked decrease in cell proliferation. When entering the decay period, the Y value continuously decreased and fell to 0.08 on the 113th day, then remaining stable at about 0.07. Within this period, the photosynthetic pigments greatly decreased, and the photosynthetic capacity was almost lost. Thus, a large number of cells died due to a lack of energy supply.

### 2.2. Production of β-Cyclocitral

#### 2.2.1. Variations of Intracellular and Extracellular Concentrations of β-Cyclocitral

**Figure 2:**
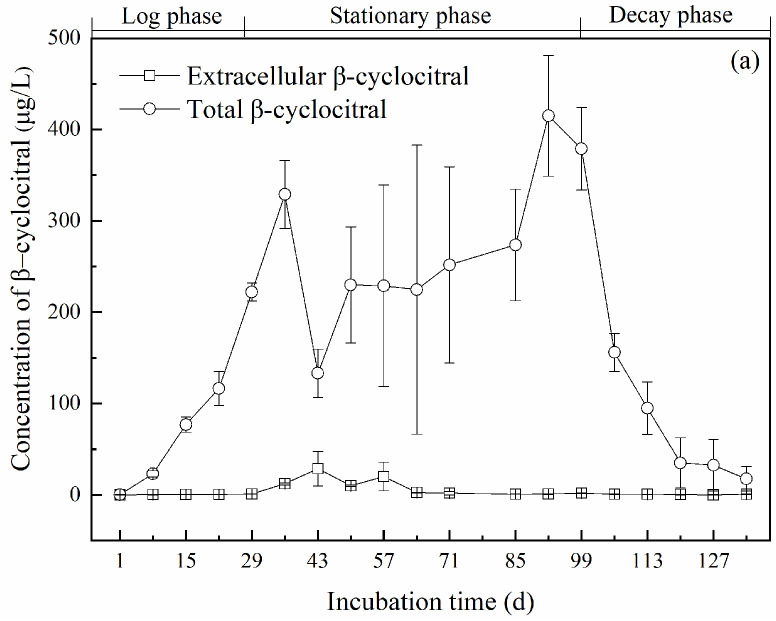

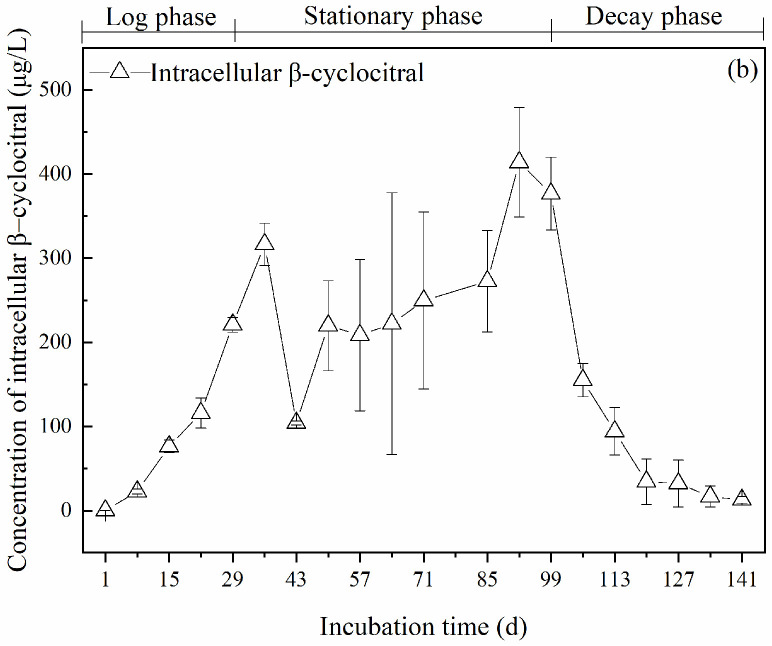
Concentration of β-cyclocitral produced by *Microcystis aeruginosa* cells during incubation: (**a**) total and extracellular concentrations of β-cyclocitral, (**b**) intracellular concentrations of β-cyclocitral.

The concentration of β-cyclocitral during the incubation time is shown in Figure 2. In the logarithmic period, the total β-cyclocitral concentration increased rapidly, from the initial value of 0.41 μg/L to 222 μg/L on the 29th day (Figure 2a). The total β-cyclocitral concentration fluctuated greatly in the early stage of the stationary period, in which it reached the peak value of 329 μg/L on the 36th day and then sharply decreased to 133 μg/L on the 43rd day. During the late stage of the stationary period, the total β-cyclocitral concentration increased gradually with the growth rate (Supplementary Figure S1), reaching the maximum value of 415 μg/L on the 92nd day. While entering the decay period, the total β-cyclocitral concentration decreased rapidly, and the lowest concentration of 13.12 μg/L was observed on day 141.

As shown in Figure 2a, the extracellular concentration of β-cyclocitral was low, accounting for less than 5% of the total concentration (except on the 43rd and 57th day). In the log phase, the extracellular β-cyclocitral concentration was always lower than 1 μg/L, although the intracellular β-cyclocitral concentration (Equation (1)) increased by 762 times, as shown in Figure 2b. It suggested that β-cyclocitral was synthetized and stored within *M. aeruginosa* cells.

In the early stage of the stationary phase (29th–57th day), the intracellular concentration of β-cyclocitral increased to 317 μg/L on the 36th day and then decreased sharply to 104 μg/L on the 43rd day (Figure 2b). The extracellular concentration of β-cyclocitral increased greatly and reached the maximum value of 28.79 μg/L on the 43rd day (Figure 2a). During the period from the 29th to the 43rd day, the decrease in both total and intracellular concentrations of β-cyclocitral was synchronized with the rise of the *P*_md_ value, which suggested the release of β-cyclocitral due to the death of algal cells and its subsequent evaporation, since no significant increase in extracellular β-cyclocitral was observed.

In the late stage of the stationary phase (57th–99th day), the intracellular concentration of β-cyclocitral gradually increased and reached the highest value of 414 μg/L on the 92nd day. The extracellular concentration of β-cyclocitral experienced a rapid decrease, reached 2.64 μg/L on the 64th day, and then remained below 2 μg/L. During this period, the algae cells grew and multiplied using the nutrients released by the dead cells in the early stage of the stationary phase. Consequently, the intracellular concentration of β-cyclocitral increased again, while the extracellular concentration remained at a low level. This indicated that during the late stage of the stationary phase, no substantial abrupt cell death occurred, which prevented the release of large amounts of intracellular β-cyclocitral from *M. aeruginosa*. It was also found that the dead cells would not be lysed within a short time and could still be detected by flow cytometry, and thus the *P*_md_ value remained stable.

In the decay period, the intracellular concentration of β-cyclocitral decreased rapidly from 377 μg/L on the 99th day to 155 μg/L on the 106th day, which was followed by a continuous reduction. The intracellular concentration of β-cyclocitral was only 12.82 μg/L on day 141, while the extracellular concentration remained below 1 μg/L. Although the *P*_md_ value was maintained at about 69%, the intracellular β-cyclocitral concentration was drastically reduced.

#### 2.2.2. Quota of β-Cyclocitral Produced per Membrane-Intact Cell

**Figure 3:**
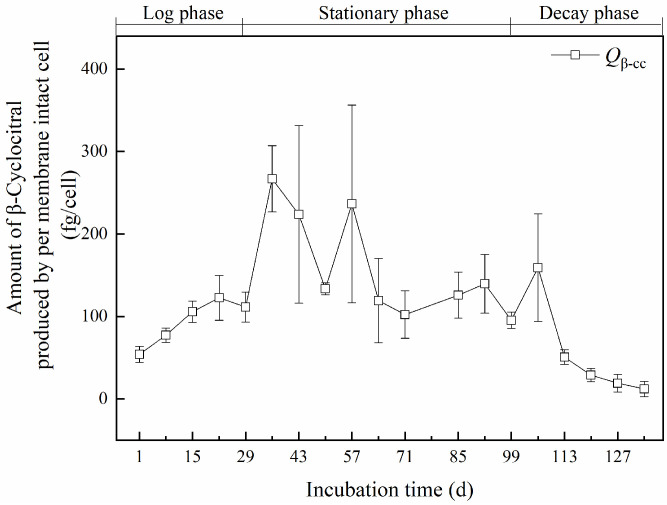
Cellular production quota (*Q*_β-cc_) of *Microcystis aeruginosa* cells during the incubation.

Intracellular β-cyclocitral was produced entirely by cells with an intact membrane. The cellular production quota (*Q*_β-cc_) was calculated by applying the Equation (2) given in Section 5.6. The results are shown in Figure 3.

In the logarithmic period, a limited increase in *Q*_β-cc_ was observed. The value was 2.2 fg/cell on the first day and increased to 4.9 fg/cell on the 22nd day. Then, it decreased slightly to 4.4 fg/cell on the 29th day. In the early stage of the stationary phase, *Q*_β-cc_ increased significantly and reached the maximum value of 10.7 fg/cell on the 36th day. Then, it fluctuated between 5.3 and 9.5 fg/cell, till the 57th day. In the late stage of the stationary phase, *Q*_β-cc_ decreased rapidly to 4.8 fg/cell on the 64th day and then remained stable with an average value of 4.7 fg/cell. In the decay period, *Q*_β-cc_ rapidly decreased reaching values below 1 fg/cell.

### 2.3. Variation of β-Cyclocitral along with β-Carotene

**Figure 4:**
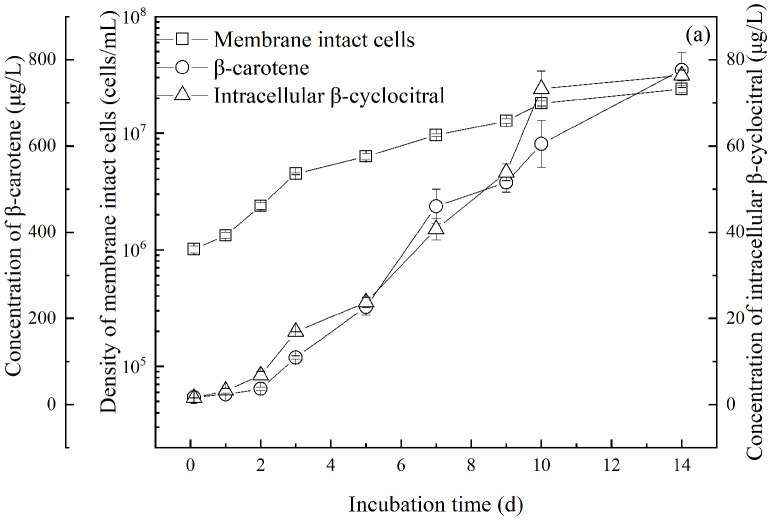

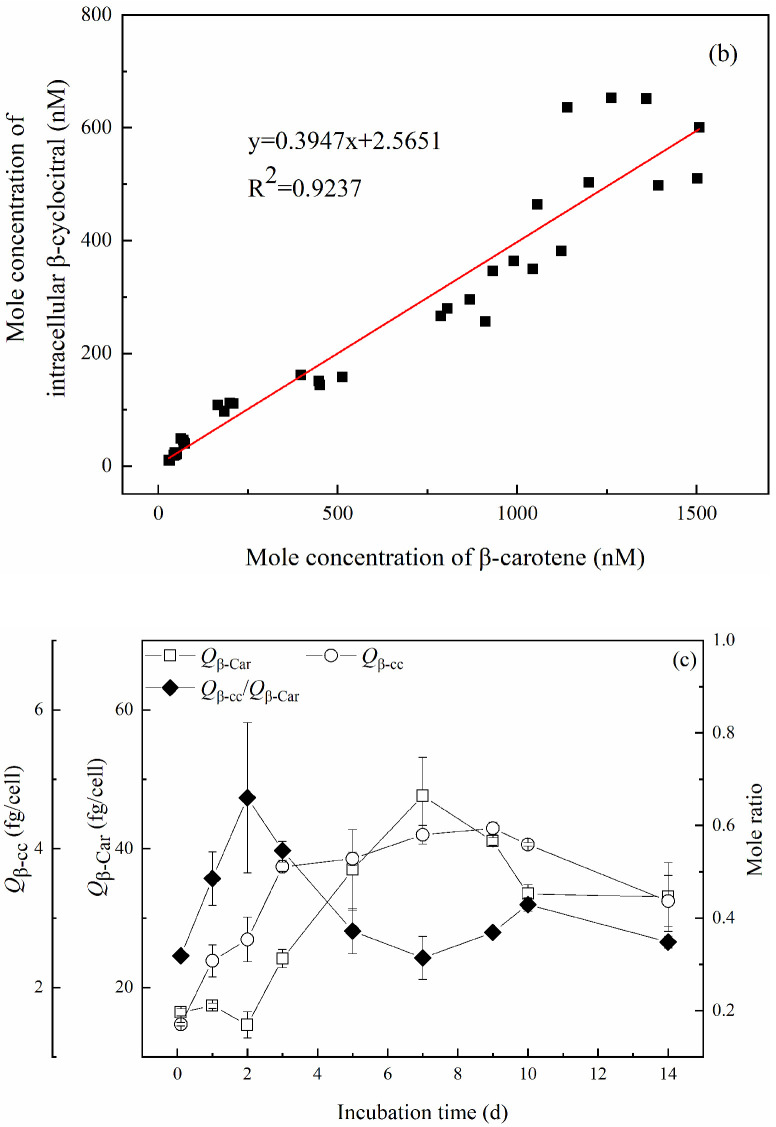
Variation of β-cyclocitral along with β-carotene in *Microcystis aeruginosa* cells during incubation. (**a**) Variations of cell density and intracellular concentrations of β-cyclocitral and β-carotene during 14 days, (**b**) relationship between intracellular β-cyclocitral and β-carotene, (**c**) variations of quotas of β-cyclocitral (*Q*_β-cc_) and β-carotene (*Q*_β-Car_) and their molar ratios.

In order to investigate the relationship between β-cyclocitral and its precursor β-carotene, a 14-day experiment with daily sampling was carried out, and the results are shown in Figure 4. Cell density and *P*_md_ were measured during the 14 days of incubation, and the density of membrane-intact cells was calculated.

The density of membrane-intact cells increased from an initial value of 1.0 × 10^6^ cells/mL to 2.4 × 10^7^ cells/mL (nearly 24 times higher) on the 14th day. The concentration of β-carotene increased from an initial value of 17.2 μg/L to 776 μg/L (nearly 45 times higher) on the 14th day. Meanwhile, the intracellular concentration of β-cyclocitral also increased sharply from an initial value of 1.5 μg/L to 76.4 μg/L (nearly 50 times higher) on the 14th day. This indicated that β-cyclocitral had a similar increasing trend to cell growth.

In order to investigate the amount of β-cyclocitral produced from a unit of β-carotene, the intracellular molar concentration of β-cyclocitral was plotted against that of β-carotene, as shown in Figure 4b. A good liner relationship with an R^2^ of 0.92 was obtained between the intracellular molar concentrations of β-cyclocitral and β-carotene. In addition, it can be seen in the figure that the molar ratio of β-cyclocitral to β-carotene was about 0.39, i.e., 1 mol β-carotene produced 0.39 mol β-cyclocitral. However, theoretically, 1 mol of β-carotene is completely transformed producing only β-cyclocitral or β-ionone, and 2 mol of β-cyclocitral or β-ionone can be obtained. In this study, 0.39 mol β-cyclocitral and 1.5 × 10^−4^ mol β-ionone (Figure S3 in the Supplementary Materials) were obtained.

The yield quotas of β-cyclocitral (*Q*_β-cc_) and β-carotene (*Q*_β-Car_) were calculated by applying the Equations (2) and (3), respectively given in Section 5.6, as shown in Figure 4c. Although both *Q*_β-Car_ and *Q*_β-cc_ increased within the first several days, variations were unsynchronized. *Q*_β-Car_ was low and stable within the first 2 days. Starting on day 3, *Q*_β-Car_ rose rapidly, reached the maximum of 47.6 fg/cell on day 7, and then gradually decreased to 33.1 fg/cell on the 14th day. In contrast, *Q*_β-cc_ increased rapidly from 1.5 fg/cell to 3.7 fg/cell within 3 days. Then, the rise became slow from day 3 to day 9, and the value finally reached the maximum of 4.3 fg/cell on day 9. *Q*_β-cc_ then decreased to 3.3 fg/cell on day 14.

## 3. Discussion

*M. aeruginosa* can produce several kinds of odorous compounds including β-cyclocitral and β-ionone. In this study, the intracellular concentration of β-cyclocitral was several tens to hundreds of times as high as the concentration of β-ionone (Figure S3 in the Supplementary Materials). The microalgal culture condition and sampling operation were strictly sterile. No thiol sulfide was detected, which might be produced during algal debris decomposition. Therefore, this study concerned β-cyclocitral as the main odorous compound produced by *M. aeruginosa* cells. The concentration of β-cyclocitral produced in different growth phases was determined at both population and single-cell levels, and the time window with the highest concentration was also determined. In addition, the cellular β-cyclocitral and β-carotene quotas were compared to assess their synchronicity, as well as the potential and efficiency of β-cyclocitral production.

The variations of the intracellular concentration of β-cyclocitral at both population and single-cell levels were similar: There was a rapid increase in the log phase, and peak values were reached in the stationary phase (Figure 2 and Figure 3). Therefore, the stationary phase is more dangerous for β-cyclocitral production and deserves closer monitoring. A previous study also indicated that the highest total β-cyclocitral concentration was measured between the stationary phase and the decay phase [26].

The total concentration of β-cyclocitral during the stationary phase varied from 133 to 415 μg/L, i.e., it was much higher than that in natural waters but lower than that in surface scum samples (Table 1). The cell density in the present study remained above 10^7^ cells/mL and even increased to 1.3 × 10^8^ cells/mL, significantly higher than the normal cyanobacterial biomass in lakes and reservoirs. In contrast, the cell density in surface scum may be as high as 10^9^ cells/mL and even higher [29]. A large cyanobacterial biomass leads to a high β-cyclocitral concentration. In addition, surface scum contains higher concentrations of extracellular polymeric substances, which are more conducive to β-cyclocitral production than water. The *Q*_β-cc_ of *M. aeruginosa* during the stationary phase fluctuated between 3.81 and 10.67 fg/cell, as indicated in previous reports. It should be noted that both total and intracellular β-cyclocitral concentrations and *Q*_β-cc_ during the stationary phase varied significantly, much more than in other growth phases. Since the beginning of the early stationary stage, the growth rate was markedly reduced (Supplementary Figure S1), and the percentage of dead cells jumped to 60%, then varying significantly. The drastic change of *M. aeruginosa* cell state in the stationary phase is probably an important reason for the fluctuation of total and intracellular β-cyclocitral concentrations and *Q*_β-cc_.

Interestingly, the times at which the intracellular concentration of β-cyclocitral at both population and single-cell levels peaked were not synchronized in this study. The peak time for the cellular quota *Q*_β-cc_ was observed in the early stage of the stationary phase (Figure 3), from day 29 to day 57, and occurred much earlier than in the population, where it peaked in the late stage of the stationary phase (Figure 2), from day 92 to day 99. Therefore, the early stage of the stationary phase is very important, and the sharp increase in the cellular quota *Q*_β-cc_ during this stage is valuable for anticipating the subsequent increase in intracellular β-cyclocitral concentration. Photosynthetic activity, with a marked drop between the early and the late stage of the stationary phase (Figure 1b), might be an indicator of β-cyclocitral concentration increase.

The increases in the cellular quotas of β-cyclocitral and β-carotene were not synchronized (Figure 4), suggesting that the production of β-cyclocitral in *M. aeruginosa* cells was affected by β-carotene, carotene oxygenase, and intracellular ROS; the dominant factor varied within different growth phases. Usually, β-cyclocitral is derived from the oxidation of β-carotene that can be catalyzed by either an enzymatic pathway involving carotene oxygenase [14,15] or a non-enzymatic pathway involving reactive oxygen species (ROS), especially singlet oxygen [16]. In this study, the intracellular molar concentrations of β-cyclocitral and β-carotene showed a good linear relationship (Figure 4b), consistently with a previous study [26], in which the essential role of β-carotene in β-cyclocitral production was confirmed. However, the increase in *Q*_β-cc_ occurred two days earlier than that of *Q*_β-Car_ (Figure 4c). In addition, the molar ratio of *Q*_β-cc_ to *Q*_β-Car_ rapidly increased within the initial two days of the log phase, and was much faster than that of *Q*_β-cc_ and *Q*_β-Car_ (Figure 4c). The results showed that the efficiency of β-cyclocitral synthesis from β-carotene in the log phase was higher than that in the subsequent periods. During the stationary phase, approximately from day 5 to day 14, a faster increase of *Q*_β-Car_ was recorded, which suggests the presence of a sufficient supply of β-carotene in *M. aeruginosa* cells. However, the decrease in the molar ratio of *Q*_β-cc_ to *Q*_β-Car_ indicated a decline in the synthetic efficiency of β-cyclocitral from β-carotene. Such decrease might be due to the shortage of carotene oxygenase. In addition, the dramatic decrease in photosynthetic activity (Figure 1b) could result in the rise of ROS (Supplementary Figure S4) and lead to the formation of β-cyclocitral [32,33], which might contribute to the temporary increase in the molar ratio of *Q*_β-cc_ to *Q*_β-Car_ during the period from day 7 to day 10 (Figure 4c).

## 4. Conclusions

Variations of β-cyclocitral concentrations in different growth phases of the cyanobacteria *M. aeruginosa* were investigated, and the synchronicity of the production of β-cyclocitral and of its precursor β-carotene at both population and single-cell levels was assessed. The intracellular concentration of β-cyclocitral (*C*_β-cc_), as well as its cellular quota (*Q*_β-cc_), increased synchronously during the log phase. However, they reached the maximum values in the late stage and early stage of the stationary phase, respectively. The stationary phase is thus more dangerous for β-cyclocitral production and deserves closer monitoring. The sharp increase in *Q*_β-cc_ in the early stationary phase is valuable for anticipating a subsequent increase in *C*_β-cc_. *C*_β-cc_ was linearly correlated with the intracellular concentration of β-carotene (*C*_β-Car_); however, the increase in *Q*_β-cc_ was unsynchronized with that of β-carotene, *Q*_β-Car_, suggesting that the production of β-cyclocitral was β-carotene-dependent in the log phase and carotene oxygenase-limited in the stationary phase.

## 5. Materials and Methods

### 5.1. Microorganism

The tested alga *M. aeruginosa* (FACHB 905) was obtained from the Freshwater Algae Culture Collection of the Institute of Hydrobiology (FACHB Collection; Wuhan, China) and cultured in BG-11 medium [34]. Cultures were incubated at 25 ± 1 °C in an algal incubation chamber (YiHeng, Shanghai, China) with a 1500 lx irradiance (fluorescent lamps; Philips, China) and a light/dark cycle of 12 h/12 h. The cultures were shaken or 3 times per day. A 141-day-long experiment was carried out to investigate the production of β-cyclocitral during a whole growth phase at the population level, and the initial cell density was 1.4 × 10^5^ cells/mL. Another 14-day-long experiment was conducted to verify the production of β-cyclocitral during the log phase and the early stage of the stationary phase. The relationships between odorous compounds and their precursors were also studied, and the initial density was set at 1.0 × 10^6^ cells/mL.

### 5.2. Flow Cytometric Analysis

A flow cytometer (FACS-Calibur, Becton Dickinson, Franklin Lakes, NJ, USA) was employed to determine cell density, membrane integrity, and oxidative stress (Supplementary Text S1). The algal cell density was determined by adding Caltag counting beads (Life Technologies, Frederick, MD, USA) [33]. Double staining with SYBR-green I (Sigma-Aldrich Corp., St. Louis, MO, USA) and propidium iodide (Life Technologies, Frederick, MD, USA) was conducted to examine the membrane integrity, which was expressed as the percentage of membrane-damaged cells (*P*_md_) [35]. Intracellular oxidative stress was characterized as the content of intracellular reactive oxygen species (ROS) and was determined by adding 2, 7-dichlorodihydrofluorescein diacetate (H_2_DCFDA, Sigma-Aldrich Corp., St. Louis, MO, USA) to the cells. All measurements were performed on the flow cytometer as described in a previous study [35].

### 5.3. Photosynthetic Activity

The photosynthetic activity, ‘Y’ value, was used to characterize the efficiency of the photosynthetic system II (PS II) in microalgal cells [36]. The photosynthetic activity of *M. aeruginosa* cells was detected by a phytoplankton pulse-amplitude-modulated fluorometer (PHYTO-PAM, Walz, Effeltrich, Germany) [37].

### 5.4. Quantification of Odorous Compounds by HS-SPME–GC–MS

For the quantification of odorous compounds including β-cyclocitral and β-ionone, headspace solid-phase microextraction (HS-SPME) coupled with GC (7890B, Agilent Technologies, Santa Clara, CA, USA) and MS (5977A, Agilent Technologies, Santa Clara, CA, USA) was used [38]. The detailed method can be found in Supplementary Text S2 and Table S1.

### 5.5. Quantification of β-Carotene by HPLC

β-carotene concentration was quantified with a high-performance liquid chromatographer (HPLC, 20-AT, SHIMADZU, Kyoto, Japan). For the determination of intracellular β-carotene concentration, a solid-phase extraction device was used after breaking the cells by liquid nitrogen freezing–thawing and homogenizer (AS ONE, Osaka, Japan) grinding [39]. The detailed method can be found in Supplementary Text S3.

### 5.6. Data Analysis

Four replicates were examined at each experimental point. The data were processed and graphed using Origin 8.5 software (OringinLab, Northampton, MA, USA). Two-factor analysis of variance was conducted using SigmaStat 3.5, and the statistical *p* value was 0.05. Except for cell density and cell membrane integrity, other flow cytometric indicators were analyzed by the normalization method and the partitioning method (i.e., separating fluorescence intensity into a weakening zone M1, a normal zone M2, and an enhancement zone M3, according to the controlled groups, and recording the proportion of each zone [38]).

The intracellular concentration of β-cyclocitral (*C*_β-cc_), the amount of β-cyclocitral produced per membrane-intact cell (*Q*_β-cc_), the amount of β-carotene produced per membrane-intact cell (*Q*_β-Car_), and the growth rate (GR) were calculated by the following equations.

*C*_β-cc_(μg/L) = total concentration − extracellular concentration, (1)

*Q*_β-cc_(fg/cell) = *C*_β-cc_/(membrane intact cell density), (2)

*Q*_β-Car_(fg/cell) = (the concentration of β-carotene)/(membrane intact cell density) (3)

GR(d^−1^) = [ln(cell density at time j) − ln(cell density at time i)]/(j − i), (4)

## Figures and Tables

**Table 1:** Summary of β-cyclocitral concentrations and *Q*_β-cc_ in different waters.

Samples	β-cyclocitral Concentrations (μg/L)	*Q*_β-cc_ (fg/Cell)	Ref
Laboratory samples	–	7.15–11.72	[14]
Laboratory samples	2000	41–865	[24]
Surface scum	1400	3.3	[20]
Fish pond	1.94 ± 1.26	-	[21]
Lake Taihu	0.35 (0.01) ^1^	-	[22]
Lake Taihu	1.37 (0.01–0.28) ^1^	-	[23]
Lake Taihu	0.15–12 (14.4–342) ^2^	-	[24]
Lake Chaohu	~0.75 ^3^	-	[30]
Source water	0.06–2.12	<0.21	[31]

^1^ Dissolved β-cyclocitral (particle-bound β-cyclocitral). ^2^ Values in the parenthesis represent β-cyclocitral concentrations in sediment, μg/kg dry weight of sediment. ^3^ Particle-bound β-cyclocitral concentrations (>97% of the sum of dissolved and particle-bound concentrations in this study).

## Data Availability

Data presented in this study are available on request from the corresponding author.

## Supplementary Materials

The following are available online at https://www.mdpi.com/article/10.3390/toxins14030201/s1, Text S1: Flow cytometric analysis, Text S2: Detailed detection method of odorous compounds using HS-SPME–GC–MS, Text S3: Detailed detection method of β-carotene using HPLC, Figure S1: Growth rate of *M. aeruginosa* during incubation, Figure S2: Photosynthetic pigments (chlorophyll, a1, a2; phycocyanobilin, b1, b2) of *Microcystis aeruginosa* cells during incubation. (partitioning analysis (a1, b1) and normalization analysis (a2, b2)), Figure S3: Variation of β-ionone concentration along with β-carotene concentration in *Microcystis aeruginosa* cells during incubation. (a). Variation of cell density and concentrations of β-ionone and β-carotene during 14 days. (b). Relationship between β-ionone and β-carotene concentrations, Figure S4: Variation of oxidative stress in *Microcystis aeruginosa* cells during incubation. Table S1: Comparison between NaCl addition and freeing–thawing for β-cyclocitral detection. References [35,38] are cited in the Supplementary Materials.
