# Supplementary material for: Production of β-Cyclocitral and Its Precursor β-Carotene in Microcystis aeruginosa: Variation at Population and Single-Cell Levels
Source: Toxins (Basel). 2022 Mar 9;14(3):201. doi: 10.3390/toxins14030201 (PMC8955627; doi:10.3390/toxins14030201)
Supplement: Supplementary file 1 [file toxins-14-00201-s001.zip › toxins-1602271-supplementary.pdf]

Supplementary Materials: Production of β-Cyclocitral and Its Precursor β-Carotene in *Microcystis aeruginosa*: Variation at Population and Single-Cell Levels

Xuejian Wang, Yinjie Zhu, Delin Hou, Fei Teng, Zhonghua Cai and Yi Tao

Text S1. Flow cytometric analysis.

Cell density was measured using a flow cytometer (FACS-Calibur, Becton Dickinson, NJ, USA) and determined by adding Caltag counting beads (Life Technologies, Frederick, MD, USA) [35]. In present work, 480 μL algal solution was blended with 20 μL counting beads, and cell density was calculated according to the following equation.

$$\text{Cell density} = (\text{cell counts/beads counts}) \times D_{beads} \times (V_{beads}/V_{algae}) \quad (1$$

Where, D_{beads} represents beads density (mL^{-1}), 10^6 mL^{-1} in this study; V_{beads} represents the volume of counting beads (μL), 20 μL in this study; V_{algae} represents the volume of algal solution (μL), 480 μL in this study.
Membrane integrity was measured with double staining with SYBR-green I (Sigma-Aldrich Corp., St. Louis, MO, USA) and propidium iodide (PI, Life Technologies, Frederick, MD, USA) [35]. SYBR-green I stains both live and dead cells, while PI can stain cells with disintegrated membrane. Membrane integrity represented as P_{md} was calculated according to the following equation.

$$P_{md} = F_{PI}/(F_{SYBR\text{-}green\ I} + F_{PI}) \quad (2$$

Where, F_{PI} represents the cell counts with PI fluorescence; $F_{SYBR\text{-}green\ I}$ represents the cell counts with SYBR-green I fluorescence.

Text S2. Detailed detection method of odorous compounds using HS-SPME-GC-MS [38].

An aliquot of 25 mL sample was placed in a 40 mL headspace vial containing 7.5 g NaCl (Sigma-Aldrich Corp., St. Louis, MO, USA). 2-Isobutyl-3-methoxypyrazine (IBMP, Sigma-Aldrich Corp., St. Louis, MO, USA) was added as an internal standard. A 50/30 μm DVB/CAR/PDMS fiber (75 μm, Supelco, Bellefonte, PA, USA) was used to extract odorous compounds and the extraction procedure lasted for 30 mins at 65 °C. Then the fiber was desorbed in the injector of the GC for 3 min at 250 °C. Helium (99.999%) was used as carrier gas and the flow rate was 1.5 mL/min. Chromatographic column was DB-5MS (30 m × 0.25 mm × 0.25 μm, Agilent Technologies, Santa Clara, CA, USA) and the column temperature program was as follows: initial temperature was 55 °C, holding for 3 min; ramp to 110 °C at a rate of 8 °C/min, holding for 2 mins at 110 °C; ramp to 280 °C at a rate of 15 °C/min. Detector temperature was 230 °C.
The selected ion monitoring (SIM) mode was chosen and the mass-nucleus ratio (m/z) was 137, 152, and 81 for β-cyclocitral, and 177, 91 and 135 for β-ionone. An aliquot of 1 mL algal culture with NaCl addition was used to determine the total β-cyclocitral and β-ionone concentrations, including both that in algal cells and that in solution, and 5 mL filtrate of algal culture with 0.45 μm PVDF films (JET Inc., Guangzhou, China) for extracellular β-cyclocitral and β-ionone concentrations. Intracellular concentrations of β-cyclocitral were the difference between total and extracellular concentrations.
Standard curves for β-cyclocitral (Sigma-Aldrich Corp., St. Loius, MO, USA) were $y = 0.0111x + 0.0405$ with an R^2 of 0.9994 (1-200 ng/L) and $y = 10.248x - 0.4146$ with an R^2 of 0.9992 (0.25-5 μg/L); that for β-ionone (Sigma-Aldrich Corp., St. Loius, MO, USA) was $y = 0.0192x + 0.0357$ with an R^2 of 0.9997 (1-200 ng/L), where y represented peak area ratio of standard β-cyclocitral or β-ionone to the internal standard and x represented β-cyclocitral or β-ionone concentration.

The addition of NaCl is to rupture and accelerate the emission of β-cyclocitral. However, the osmotic shock caused by NaCl may activate the carotene oxygenase, leading to the production of β-cyclocitral, which could affect the detecting results. To clarify the effect of NaCl, two different pretreatment methods (one was NaCl addition, the other was freeze thawing method) were applied to the same cell culture. The detecting result was presented below and it revealed that adding NaCl didn't increase the amount of β-cyclocitral compared with freeze thawing method. In fact, the detected concentration of β-cyclocitral using NaCl addition method was lower than freeze thawing method. Yet the pretreatment method in the present study can avoid the emission of β-cyclocitral during freeze thawing process.

Table S1. Comparison between NaCl addition and freeze thawing in β-cyclocitral detection.

Pretreatment method	Detected concentration (μg/L)		
NaCl addition	3.78	3.76	3.05
Freeze thawing	122.54	192.5	183.96

Text S3. Detailed detection method of β-carotene using HPLC.

An aliquot of 10 mL sample was centrifuged for 15 min under 11,000 rpm and 4 °C. Supernatant was discarded and cell pellets were frozen in liquid nitrogen, followed by grinding for at least 10 min using a homogenizer (AS ONE, Osaka, Japan). PBS was added to re-suspend the broken cells. β-carotene was extracted using a solid phase extraction device (Supelco, Bellefonte, PA, USA) and then quantified with a high-performance liquid chromatography (20-AT, SHIMADZU, Kyoto, Japan). The mobile phase consisted of methanol (CNW Technologies, Düsseldorf, Germany), acetonitrile (CNW Technologies, Düsseldorf, Germany) and tetrahydrofuran (Sigma-Aldrich Corp., St. Louis, MO, USA) with the volume ratio of 45:50:5 and the flow rate was 1.0 mL/min. Chromatographic column was YMC-Pack ODS-A (5 μm, 12 nm; 150×4.6 mm I.D.) (YMC, Kyoto, Japan) and the column temperature was 30 °C. The absorption of β-carotene was 455 nm. Samples were injected automatically and the injection volume was 20 μL.

Standard curve for β-carotene (CNW Technologies, Düsseldorf, Germany) was $y = 152942x - 29689$ with an R^2 of 0.9989 (0.5-12 mg/L), where y represented peak area of β-carotene and x represented β-carotene concentration.

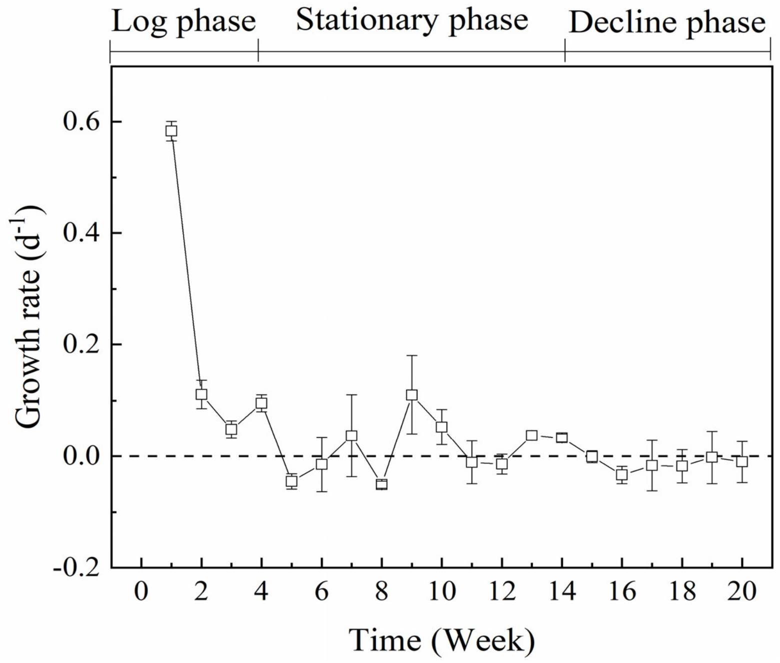

Figure S1. Growth rate of *M. aeruginosa* during incubation. Sampling was conducted once a week and thus growth rate was calculated between two consecutive sampling time points. The whole experiment lasted for a total of 20 weeks.

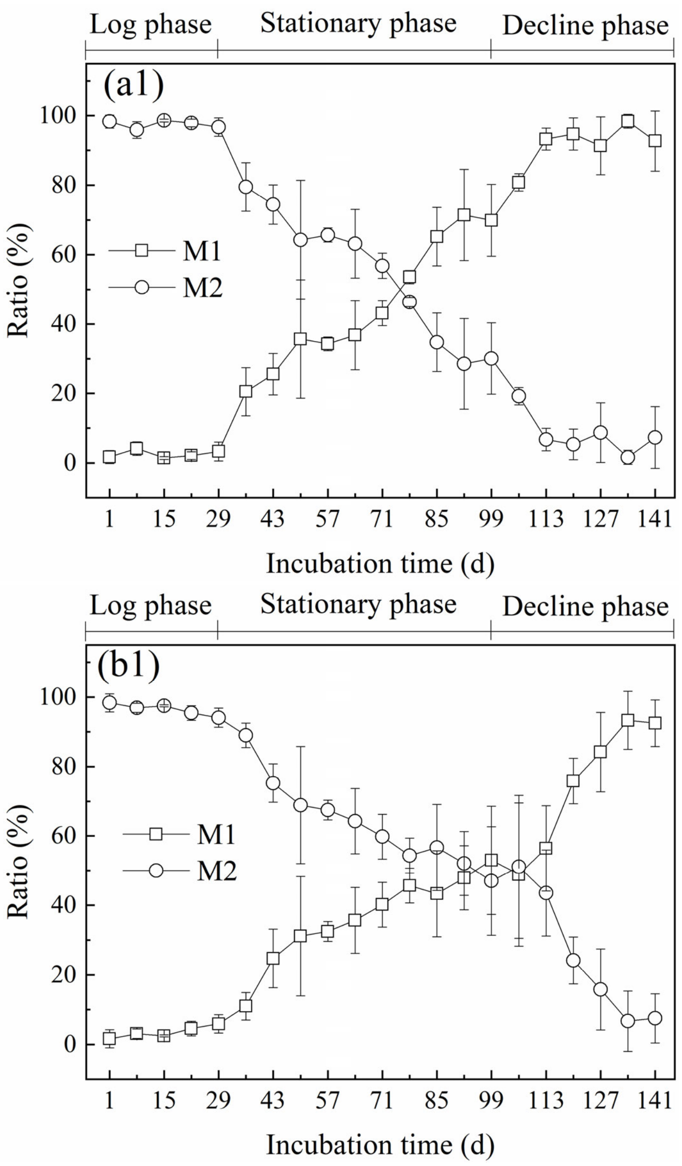
Log phase
Stationary phase
Decline phase
(a1)
Ratio (%)
M1
M2
Incubation time (d)
Log phase
Stationary phase
Decline phase
(b1)
Ratio (%)
M1
M2
Incubation time (d)

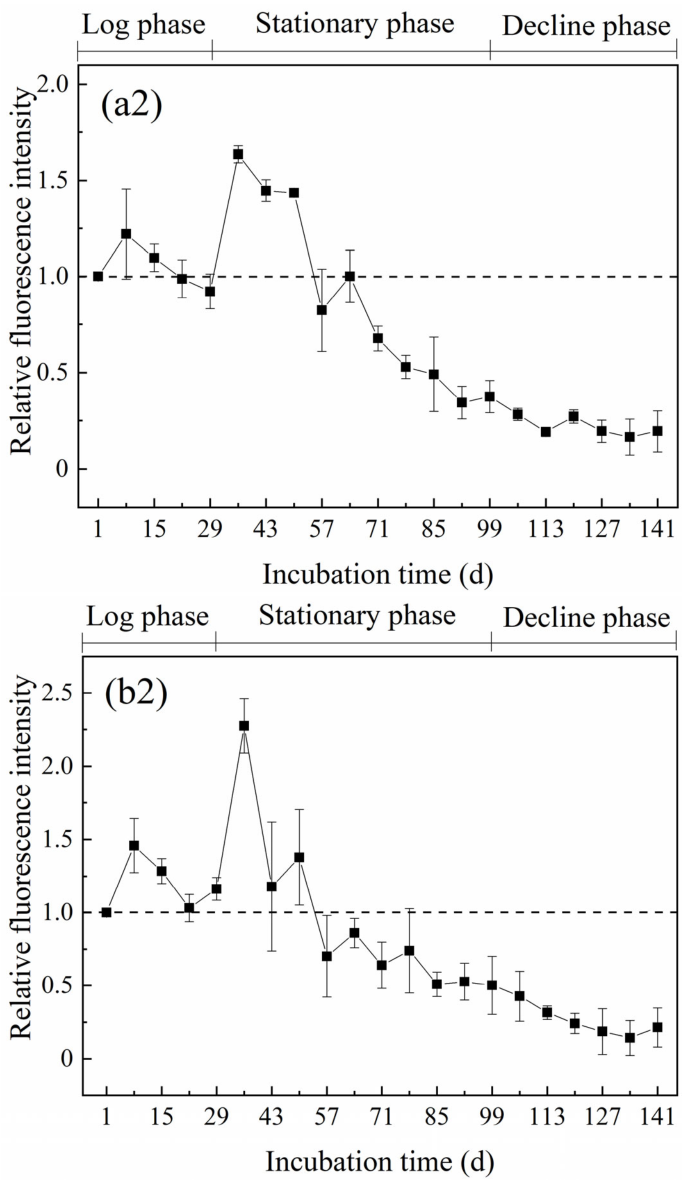

Figure S2. Photosynthetic pigments (chlorophyll, a1, a2; phycocyanobilin, b1, b2) of *Microcystis aeruginosa* cells during incubation. (partitioning analysis (a1, b1) and normalization analysis (a2, b2)).

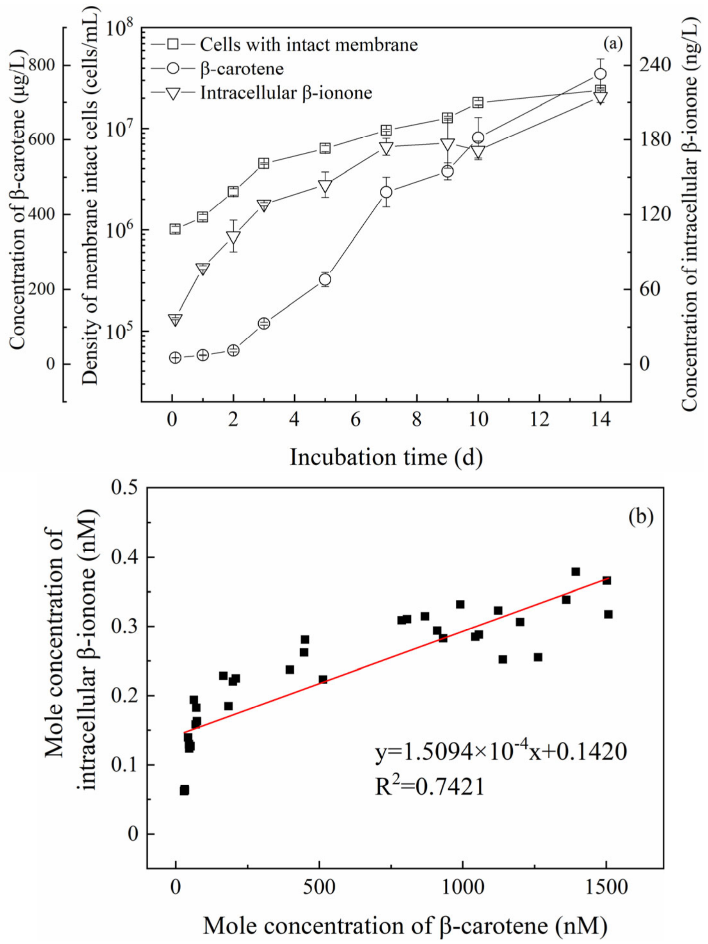

Figure S3. Variation of β-ionone along β-carotene of *Microcystis aeruginosa* cells during incubation. (a). Variation of cell density and concentrations of β-ionone and β-carotene during 14 days. (b). The relationship between β-ionone and β-carotene.

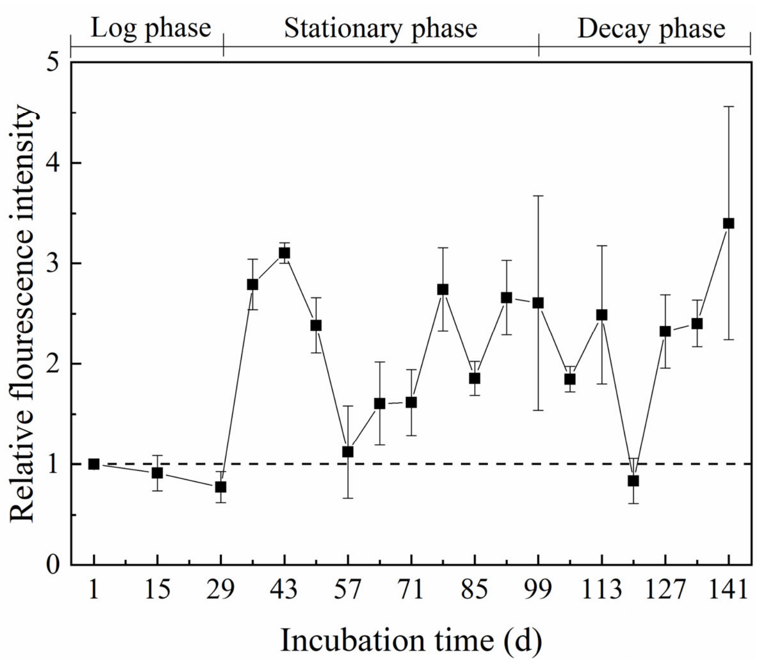

Figure S4. Variation of oxidative stress of *Microcystis aeruginosa* cells during incubation.

References

35 Tao, Y.; Zhang, X.; Au, D.W.T.; Mao, X.; Yuan, K. The effects of sub-lethal UV-C irradiation on growth and cell integrity of cyanobacteria and green algae. *Chemosphere*, **2010**, *78*, 541–547.

38 Tao, Y.; Hou, D.; Zhou, T.; Cao, H.; Zhang, W.; Wang, X. UV-C suppression on hazardous metabolites in *Microcystis aeruginosa*: Unsynchronized production of microcystins and odorous compounds at population and single-cell level. *J. Hazard. Mater.* **2018**, *359*, 281–289.
